# Disturbing Weight Cutting Behaviors in Young Combat Sports Athletes: A Cause for Concern

**DOI:** 10.3389/fnut.2022.842262

**Published:** 2022-02-02

**Authors:** Nemanja Lakicevic, Reid Reale, Giuseppe D'Antona, Emi Kondo, Hiroyuki Sagayama, Antonino Bianco, Patrik Drid

**Affiliations:** ^1^Sport and Exercise Sciences Research Unit, University of Palermo, Palermo, Italy; ^2^UFC Performance Institute, Shanghai, China; ^3^CRIAMS-Sport Medicine Centre, University of Pavia, Pavia, Italy; ^4^Faculty of Health and Sport Sciences, University of Tsukuba, Tsukuba, Japan; ^5^Japan Society for the Promotion of Science, Tokyo, Japan; ^6^Faculty of Sport and Physical Education, University of Novi Sad, Novi Sad, Serbia

**Keywords:** rapid weight loss, rapid weight gain, children, adolescents, health, performance

## Introduction

Problematic weight cutting behaviors in combat sports have been addressed in the scientific literature since the 1930s (1). Indeed, given the available evidence it may be the case that making weight/weight cycling [i.e., rapid weight loss (RWL) prior to weigh-in followed by rapid weight gain prior to competition] has been practiced in combat sports since weight divisions have been introduced. These practices have led to several fatalities (2), which occurred as a consequence of making weight rather than any sports-related injury. Unfortunately, RWL-related deaths still persist into recent times (3). Existing literature has detailed patterns of weight cycling (e.g., methods, magnitudes, frequency, and prevalence) in adult combat sport athletes across various sports, with data revealing RWL is ubiquitous with prevalence reaching 90% in some combat sports (4). However, less is known about the weight making behaviors of child and adolescent combat sport athletes.

## Alarming Trends

In the wake of the tragic deaths of three adolescent American wrestlers attempting to make weight in 1997 (2), several papers have been published on this matter in young athletes, causing concern among scientists in the field. As is the case for adult athletes, most adolescent athletes in weight-class sports compete in weight classes below their “natural” (or “walk around”) body weight (5). For example, upon examination of a large cohort (*n* = 822) of judo athletes from various regions of Brazil (4), researchers reported athletes start engaging in RWL as young as 4 years of age. Similar trends were found in Israel, where judo athletes again reported practicing RWL from as early as four (6). These findings align with previously obtained data detailed in a case study from the United States of a 5 year-old child partaking in RWL for a wrestling competition (7).

Aside from the age at which weight making may begin, of additional major concern are the methods employed by young combat athletes to achieve a desired weight. Studies have repeatedly shown that the tools utilized to achieve RWL are identical between young combat sport athletes and their adult counterparts. Specifically, the most commonly reported RWL methods are fluid and food restriction, increased levels of intense exercise, training in impermeable clothing, as well as frequent sauna use (5, 6, 8–10). Of concern, some studies reported that minor-aged combat athletes take laxatives (8, 9) and incorporate spitting (11) as part of their RWL strategy. The commonality of all these methods is the resulting temporary reduction in total body water, muscle glycogen levels and gut content (12–14).

While RWL duration differs individually and among combat sports, the mutual feature of most weight making attempts is to maximize weight loss in the last few days prior to the official weigh in (15). Likewise, the magnitude of weight loss also differs individually and may depend on the weight class, however weight loss of ~1–6% of pre-weigh in body mass is common in young combat athletes (6, 9). Prevalence of RWL in this population ranges from 25 to 80% (6, 9–11, 16), with higher prevalence in older combat athletes (94%). Notably however, the frequency of RWL in young combat sport athletes is not well-described as only one study reported that adolescent judo athletes engage 2.2 times per year in RWL (6), but RWL is likely dependent on the number of competitions one participates in (4). As a consequence of RWL, junior wrestlers reported experiencing headache, dizziness, nausea, hot flashes, nose bleeds and to a smaller degree feverish, disorientation and increased heart rate, while 30% of junior boxers experienced fatigue and 21% experienced myalgia during RWL (8, 10).

## Risks of Rapid Weight Loss for Young Athletes

With dehydration being the most common method of RWL, interest in how this method impacts youth and adolescents' physiology is warranted. Young individuals have an immature sweating function compared to adults; as such they regulate body temperature by increasing peripheral blood redistribution rather than sweating (evaporative cooling) (17). Therefore, youth athletes are more likely than adults to experience a rise in core body temperature, particularly in hot environments such as saunas, increasing the risk of heat stroke.

In addition to the concerns associated with pre-competition acute weight loss, some young athletes go even further, striving not to gain weight between competitions in order to remain in a specific weight class for a period of 2 years or longer (6), thereby suppressing natural growth and development which normally occurs during childhood and adolescence. Young athletes require enough energy and other nutrients to grow, with peak bone and height velocity occurring around the age of 12 in girls and 14 in boys, and first menses in female athletes occurring around ~14 years (18). Energy deficiency in the growth phase, not only stunts growth, but may impact additional metabolic systems and physical performance (19). Furthermore, young athletes have a particular need for micronutrients (e.g., iron, calcium, or vitamin D) due to the rapid growth experienced during this time (20).

## Discussion

To those unfamiliar with the world of combat sports, it would seem counterintuitive to intentionally deprive oneself of food and fluids prior to engaging in combat sport competition. Nevertheless, as has been repeatedly shown, RWL is highly prevalent and indeed deeply embedded in the culture of combat sports, which consequently leads to situations where athletes who may have not wanted to engage in RWL, do so in order to avoid a perceived disadvantage (21). Although evidence as to whether RWL prior to- and increased body mass (BM) at the time of competition is a key determinant of success in combat sports remains somewhat equivocal overall, BM regain post-weigh-in may be beneficial in grappling sports (e.g., judo, wrestling) where the goal is to manipulate an opponent's body and impose one's own body weight (22). Conversely, increased BM is noted to likely be less beneficial in striking sports, where success depends more on tactical executions of movement, footwork, speed, and successfully landing blows to an opponent's body (10, 23).

Data indicate that young combat athletes are strongly influenced by their coaches to engage in RWL, with very few athletes seeking advice from health professionals such as physicians or nutritionists (6, 11). This environment of influence mirrors the case in adult combat sport populations (24). In the absence of evidenced based education programs or professional intervention, it is evident that RWL practices are routinely passed from coach to athlete, and from athlete to athlete, with such practices centered on tradition rather than well-informed evidenced based methods (6). It is of particular concern that individuals with poor nutrition and medical understanding are advising athletes to partake in RWL (25), even when it is recognized these practices can result in acute and chronic complications ranging from mild, transient health disturbances to substantial performance decrements and even death.

Optimal nutrition is particularly important during childhood and adolescence, when dramatic changes in fat-free mass occur, which when combined with physical activity requires a substantial intake of energy alongside protein and other nutrients (26). Body size adjusted metabolic rate is the highest in infants, which then decline by about 3% per year until reaching adult levels at ~20 years of age (27). Indeed, absolute energy requirements in humans are at their highest during the late teens, declining slightly thereafter, and then remaining constant until the 50s. Junior athletes, like adults, may experience chronic low energy availability (LEA) resulting in relative energy deficiency, acutely and chronically impacting training and daily physiological functions such as menstruation, bone health, and lipid profiles (28). Interestingly, bone mineral density in wrestling and judo athletes who began RWL practices as juniors is higher than that of the general population (29). This may suggest the exposure to weight bearing activity and physical impacts typical of combat sports may have a larger effect on bone mineral density than that of repeated LEA. Note these findings do not allow one to conclude that chronic LEA does not impact bone mineral density, only that repeated episode of RWL may not be—in this specific context. Chronic LEA in junior athletes may still have significant consequences on bone metabolism, however further research needs to be done, as the effect of temporary RWL, combined with persistent LEA and weight cycling in juniors across the life span has not undergone detailed investigation.

The issue of RWL in combat sports as a whole is still a contentious one, although evolution in the literature and recommendations to curtail harmful practices continue (3). In adult athletes, modern guidelines have taken a pragmatic approach which aim to balance athletes' health and safety with their desire to recognize real or perceived benefits from acute and chronic BM manipulation (12, 30). While these guidelines may suitably strike the correct balance in adult populations, additional considerations are warranted in regards to youth athletes. Until further research answers pertinent questions and revised guidelines for youths are published, parents, practitioners, coaches, and adult athletes should take a conservative approach when discussing potential BM loss or management strategies with young athletes whenever possible.

## Author Contributions

NL, RR, EK, and HS contributed to conception and design of the study and wrote the first draft of the manuscript. NL, GD'A, AB, PD, and RR wrote sections of the manuscript. All authors contributed to manuscript revision, read, and approved the submitted version.

## Conflict of Interest

The authors declare that the research was conducted in the absence of any commercial or financial relationships that could be construed as a potential conflict of interest.

## Publisher's Note

All claims expressed in this article are solely those of the authors and do not necessarily represent those of their affiliated organizations, or those of the publisher, the editors and the reviewers. Any product that may be evaluated in this article, or claim that may be made by its manufacturer, is not guaranteed or endorsed by the publisher.

## References

[1] Kenney HE. The problem of weight making for wrestling meets. J Health Phys Educ. (1930) 1:24–49. 10.1080/23267240.1930.10623427

[2] Remick D Chancellor K Pederson J Zambraaki EJ Sawka MN Wenger CB. Hyperthermia and dehydration-related deaths associated with intentional rapid weight loss in three collegiate wrestlers- North Carolina, Wisconsin, and Michigan, November-December 1997. J Am Med Assoc. (1998) 47:105–8. 9480411

[3] Burke LM Slater GJ Matthews JJ Langan-Evans C Horswill CA. ACSM expert consensus statement on weight loss in weight-category sports. Curr Sports Med Rep. (2021) 20:199–217. 10.1249/JSR.0000000000000831 33790193

[4] Giannini Artioli G Gualano B Franchini E Scagliusi FB Takesian M Fuchs M. Prevalence, magnitude, and methods of rapid weight loss among judo competitors. Med Sci Sports Exerc. (2010) 42:436–42. 10.1249/MSS.0b013e3181ba8055 19952804

[5] Boisseau N Vera-Perez S Poortmans J. Food and fluid intake in adolescent female judo athletes before competition. Pediatr Exerc Sci. (2005) 17:62–71. 10.1123/pes.17.1.62

[6] Berkovich B El Eliakim A Nemet D Stark AH Sinai T. Rapid weight loss among adolescents participating in competitive judo. Int J Sport Nutr Exerc Metab. (2016) 26:276–84. 10.1123/ijsnem.2015-0196 26479490

[7] Sansone RA Sawyer R. Weight loss pressure on a 5 year old wrestler. Br J Sports Med. (2005) 39:e2. 10.1136/bjsm.2004.013136 15618326 PMC1725024

[8] Alderman BL Landers DM Carlson J Scott JR. Factors related to rapid weight loss practices among international-style wrestlers. Med Sci Sports Exerc. (2004) 36:249–52. 10.1249/01.MSS.0000113668.03443.66 14767247

[9] Viveiros L Moreira A Zourdos MC Aoki MS Capitani CD. Pattern of weight loss of young female and male wrestlers. J Strength Cond Res. (2015) 29:3149–55. 10.1519/JSC.0000000000000968 25932982

[10] Zubac D Karnincic H Sekulic D. Rapid weight loss is not associated with competitive success in elite youth olympic-style boxers in Europe. Int J Sports Physiol Perform. (2018) 13:860–6. 10.1123/ijspp.2016-0733 29182408

[11] do Nascimento MVS Reinaldo JM Brito CJ Mendes-Netto RS. Weight cutting is widespread among adolescent judoka regardless of experience level: the need of weight control and educational programs. J Phys Educ Sport. (2020) 20:150–5. 10.7752/jpes.2020.0102033809896

[12] Reale R Slater G Burke LM. Acute-Weight-Loss strategies for combat sports and applications to olympic success. Int J Sports Physiol Perform. (2017) 12:142–51. 10.1123/ijspp.2016-0211 27347784

[13] Sagayama H Yoshimura E Yamada Y Ichikawa M Ebine N Higaki Y. Effects of rapid weight loss and regain on body composition and energy expenditure. Appl Physiol Nutr Metab. (2014) 39:21–7. 10.1139/apnm-2013-0096 24383503

[14] Kondo E Sagayama H Yamada Y Shiose K Osawa T Motonaga K. Energy deficit required for rapid weight loss in elite collegiate wrestlers. Nutrients. (2018) 10:536. 10.3390/nu10050536 29701639 PMC5986416

[15] Lakicevic N Paoli A Roklicer R Trivic T Korovljev D Ostojic SM. Effects of rapid weight loss on kidney function in combat sport athletes. Medicina. (2021) 57:551. 10.3390/medicina57060551 34072641 PMC8229569

[16] Karnincic H Mario B Kristijan S. Mood aspects of rapid weight loss in adolescent wrestlers. Kinesiology. (2016) 48:229–36. 10.26582/k.48.2.721399531

[17] Falk B Dotan R. Children's thermoregulation during exercise in the heat: a revisit. Appl Physiol Nutr Metab. (2008) 33:420–7. 10.1139/H07-185 18347699

[18] Kishali NF Imamoglu O Katkat D Atan T Akyol P. Effects of menstrual cycle on sports performance. Int J Neurosci. (2006) 116:1549–63. 10.1080/00207450600675217 17145688

[19] Mountjoy M Sundgot-Borgen JK Burke LM Ackerman KE Blauwet C Constantini N. IOC consensus statement on relative energy deficiency in sport (RED-S): 2018 update. Br J Sports Med. (2018) 52:687–97. 10.1136/bjsports-2018-099193 29773536

[20] Desbrow B. Youth athlete development and nutrition. Sport Med. (2021) 51:3–12. 10.1007/s40279-021-01534-6 34515968 PMC8566439

[21] Artioli GG Saunders B Iglesias RT Franchini E. It is time to ban rapid weight loss from combat sports. Sport Med. (2016) 46:1579–84. 10.1007/s40279-016-0541-x 27102173

[22] Reale R Cox GR Slater G Burke LM. Regain in body mass after weigh-in is linked to success in real life judo competition. Int J Sport Nutr Exerc Metab. (2016) 26:525–30. 10.1123/ijsnem.2015-0359 27206272

[23] Reale R Cox GR Slater G Burke LM. Weight regain: no link to success in a real-life multiday boxing tournament. Int J Sports Physiol Perform. (2017) 12:856–63. 10.1123/ijspp.2016-0311 27834565

[24] Reale R Slater G Burke LM. Weight management practices of australian olympic combat sport athletes. Int J Sports Physiol Perform. (2018) 13:459–66. 10.1123/ijspp.2016-0553 28872383

[25] Lakicevic N Mani D Paoli A Roklicer R Bianco A Drid P. Weight cycling in combat sports: revisiting 25 years of scientific evidence. BMC Sport Sci Med Rehabil. (2021) 13:154. 10.1186/s13102-021-00381-2 34906212 PMC8670259

[26] Westerterp KR Yamada Y Sagayama H Ainslie PN Andersen LF Anderson LJ. Physical activity and fat-free mass during growth and in later life. Am J Clin Nutr. (2021) 114:1583–1589. 10.1093/ajcn/nqab260 34477824 PMC8574623

[27] Pontzer H Yamada Y Sagayama H Ainslie PN Andersen LF Anderson LJ. Daily energy expenditure through the human life course. Science. (2021) 373:808–12. 10.1126/science.abe5017 34385400 PMC8370708

[28] Loucks AB Kiens B Wright HH. Energy availability in athletes. J Sports Sci. (2011) 29 (Suppl. 1):S7–15. 10.1080/02640414.2011.588958 21793767

[29] Sagayama H Kondo E Tanabe Y Ohnishi T Yamada Y Takahashi H. Bone mineral density in male weight-classified athletes is higher than that in male endurance-athletes and non-athletes. Clin Nutr ESPEN. (2020) 36:106–10. 10.1016/j.clnesp.2020.01.008 32220352

[30] Reale R Slater G Burke LM. Individualised dietary strategies for olympic combat sports: acute weight loss, recovery and competition nutrition. Eur J Sport Sci. (2017) 17:727–40. 10.1080/17461391.2017.1297489 28316263

